# Interfacial Modulation of Graphene by Polythiophene with Controlled Molecular Weight to Enhance Thermal Conductivity

**DOI:** 10.3390/membranes11110895

**Published:** 2021-11-19

**Authors:** Ya Li, Yu Wang, Peng Chen, Ru Xia, Bin Wu, Jiasheng Qian

**Affiliations:** Key Laboratory of Environment-Friendly Polymeric Materials of Anhui Province, School of Chemistry & Chemical Engineering, Anhui University, Hefei 230601, China; c18101004@stu.ahu.edu.cn (Y.L.); c19301127@stu.ahu.edu.cn (Y.W.); chenpeng@ahu.edu.cn (P.C.); 96047@ahu.edu.cn (R.X.)

**Keywords:** poly(3-hexylthiophene), molecular weight, membrane, thermal conductivity

## Abstract

With a trend of continuing improvement in the development of electronic devices, a problem of serious heat accumulation has emerged which has created the need for more efficient thermal management. Graphene sheets (GNS) have drawn much attention with regard to heat transfer because of their excellent in-plane thermal conductivity; however, the ultrahigh interfacial thermal resistance between graphene lamellae has seriously restricted its practical applications. Herein, we describe heat transfer membranes composed of graphene which have been modified by intrinsic thermally conductive polymers with different molecular weights. The presence of macromolecular surface modifiers not only constructed the graphene heat transfer interface by π–π interactions, but also significantly enhanced the membranes’ in-plane thermal conductivity by utilizing their intrinsic heat transfer properties. Such results indicated that the in-plane thermal conductivity of the fabricated membrane exhibits a high in-plane thermal conductivity of 4.17 W m^−1^ K^−1^, which, containing the GNS modified with 6000 g/mol (M_n_) of poly(3-hexylthiophene) (P3HT), was 26 times higher that of poly (vinylidene fluoride) (PVDF). The P3HT molecular chain with specific molecular weight can form more matching structure π–π interactions, which promotes thermal conductivity. The investigation of different molecular weights has provided a new pathway for designing effective interfacial structures to relieve interface thermal resistance in thermally conductive membranes.

## 1. Introduction

With the continuous development of electronic devices towards higher power and higher density, the phenomenon of heat accumulation has occurred [1,2]. In order to ensure the normal functioning of electronic devices, more stringent requirements have been put forward for the heat dissipation performance of electronic devices [3,4]. Polymers have drawn attention in the field of thermal management because of their electrical insulation, corrosion resistance, and ease of processing [5,6,7]. The low thermal conductivity of polymers can be compensated by introducing highly thermally conductive GNS, which have been widely used in heat transfer because of their high in-plane thermal conductivity. However, the high interfacial thermal resistance between graphene sheets seriously restricted the further promotion of thermal conductivity [8,9]. Hence, the interfacial thermal resistance between fillers has always limited the application of membranes. Therefore, further interface optimization for reducing the interfacial thermal resistance between the graphene lamellae is imperative in order to further promote the thermal conductivity of graphene-based membranes.

The interfacial heat transfer performance between fillers could be improved by surface modification using covalent bonds or non-covalent bonds. The covalent modification might disrupt the lattice structure of the filler and result in phonon scattering [10]. Nevertheless, thermal conductivity could be substantially improved by non-covalent modification without destroying the filler structure. Buehler et al. [11] found that the thermal conductivity of graphene modified with a side chain of octane was more than 10% higher than that of composites modified with a side chain of butane and dodecane by molecular dynamics simulation. Andersson et al. [12,13] found that the thermal conductivity of poly (vinyl pyrrolidone) (10,000 g/mol)/multi-walled carbon nanotube (MWNT@PVP (10,000 g/mol)) composite was 3.64 W m^−1^ K^−1^, which was much greater than the MWNT@PVP (40,000 g/mol) (2.14 W m^−1^ K^−1^) and MWNT@PVP (50,000 g/mol) (2.40 W m^−1^ K^−1^) composites at the same addition amount. As a result, the molecular weight of the macromolecule would affect the thermal conductivity of the composite. However, most macromolecular modifiers show an ultralow intrinsic thermal conductivity, which significantly restricts their application with respect to improving the thermal performance of graphene-based membranes. Hence, the influence of the molecular weight and the high intrinsic thermal conductivity of the macromolecular modifier on the thermal conductivity of the composite should been considered. The thermal conductivity of poly(thiophene) could reach 4.4 W m^−1^ K^−1^—a comparatively high value in polymers [14,15]. In addition, the molecular weight of poly(thiophene) could be well controlled by the Grignard reaction method (GRIM) [16,17].

In this work, poly(3-hexylthiophene) (P3HT) with different molecular weights has been investigated as a macromolecular modifier to enhance the thermal conductivity of graphene-based membranes. The modified graphene (GNS@P3HT) fillers with four molecular weights of P3HT were successfully prepared by π–π interaction. The influence of P3HT with different molecular weights on the thermal conductivity of composites was analyzed. The conclusion has a certain guiding significance for the preparation of high thermal conductivity membranes by macromolecular modifications in the future.

## 2. Materials and Methods

### 2.1. Materials

The 2,5-dibromo-3-hexylthiophene (C_10_H_14_Br_2_S), methylmagnesium bromide (1.0 M) (CH_3_BrMg), n-hexane, tetrahydrofuran (THF), ammonium sulfate ((NH_4_)_2_SO_4_) and N,N-dimethylformamide (DMF) were purchased from Shanghai Aladdin Biochemical Technology Co., Ltd., Shanghai, China. Poly(vinylidene fluoride) (PVDF) was obtained from Alfa Aesar Chemical Co., Ltd., (Shanghai, China). Dichloro[1,3-bis(diphenylphosphino)propane] nickel (Ni(dppp)Cl_2_) was bought from Sigma-Aldrich (Shanghai, China). The chloroform and methanol were supplied by Sinopharm Chemical Reagent Co., Ltd. (Shanghai, China). Graphite foil was provided by Suzhou Graphene Nanotechnology Co., Ltd., Suzhou, China. All chemical reagents were analytical reagent grade and, except for tetrahydrofuran, used without further purification.

### 2.2. Synthesis of P3HT with Controllable Molecular Weight

The reaction mechanism of P3HT is shown in Figure S1. The 6.24 mmol of C_10_H_14_Br_2_S and 28 mL of anhydrous THF were added into a 100 mL three-necked round-bottomed flask and stirred magnetically for 10 min under the conditions of an ice bath and N_2_. The 6.864 mmol of CH_3_BrMg was added dropwise, followed by magnetic stirring for 10 min. The reaction was continued, with stirring for a further 2 h after a certain temperature was reached. After a certain amount of Ni(dppp)Cl_2_ catalyst was added, the reaction was stirred at this corresponding temperature for 2 h. Then, 20 mL of methanol was added to the reaction system to quench the reaction. After that, it was poured into 200 mL of methanol for precipitation. The resulting dark brown solid was Soxhlet extracted with methanol (to obtain unreacted 2,5-dibromo-3-hexylthiophene and salt), n-hexane (to remove the catalyst and oligomers), and chloroform, in turn. Finally, the obtained chloroform solution was concentrated, precipitated in methanol, and dried in a vacuum oven at 60 °C for 24 h to obtain a reddish-brown solid. To obtain P3HT of different molecular weights, the reaction temperature and the amount of catalyst added were not written specifically; these measures are given in the discussion of the results.

### 2.3. Preparation of GNS

GNS was prepared by electrochemical exfoliation of graphite foil, where 0.1 mol/L (NH_4_)_2_SO_4_ aqueous solution was used as the electrolyte, graphite foils as the anode and Pt flakes as the cathode. A constant voltage of 15 V was applied to the electrodes for the electrochemical exfoliation of the graphite flakes. After the graphite flake exfoliation was finished, the product was filtered through the poly (tetra fluoroethylene) (PTFE) membrane (pore size 0.1 μm), washed three times with deionized water, and lyophilized in a freeze-dryer for 24 h to obtain GNS.

### 2.4. Preparation of GNS@P3HT Filler by π–π Stacking

The homogeneously dispersed P3HT solution was gained by adding 200 mg of P3HT to 40 mL of chloroform and then stirring to dissolve. Then, 100 mg of GNS was slowly added to the P3HT solution, followed by magnetic stirring for 24 h at room temperature. The excess P3HT was removed by filtering and washed with chloroform for three times. Finally, the GNS@P3HT filler was obtained by vacuum drying at 60 °C for 24 h. For the convenience of expression, it was abbreviated as ‘GNS@P3HT(X)’, where ‘X’ represents the molecular weight of P3HT.

### 2.5. Fabrication of GNS@P3HT(X)/PVDF Membranes

The GNS@P3HT(X)/PVDF membranes were prepared by the solution blending method and the scraping method. The PVDF solution was obtained by adding 2 g of PVDF to 10 mL of DMF solution and heating at 60 °C. The homogeneous GNS@P3HT/PVDF solution was gained by adding the GNS@P3HT to PVDF solution according to a certain proportion and stirring continuously for 12 h. Then, the membrane was scraped on a clean glass plate with a thickness of 250 μm using a four-sided film applicator. Finally, the membrane was dried on a heated plate at 60 °C, and then vacuum dried at 60 °C for 24 h to obtain GNS@P3HT fillers with different additions of P3HT with different molecular weights. PVDF membranes with GNS@P3HT contents of 0%, 1%, 5%, 10%, 20%, and 25%, respectively, were developed according to the above method. The membranes were abbreviated as ‘YGNS@P3HT(X)/PVDF’, where ‘X’ and ‘Y’ denoted the molecular weight of P3HT and the mass fraction of the added filler, respectively. For comparison, YGNS/PVDF was obtained using the same method.

According to the above method, PVDF membranes with 25 wt% of GNS or GNS@P3HT were not successfully prepared, because the addition of 25% filler made the solution lose its fluidity so that it could not be scraped (see Figure S2). 

### 2.6. Characterization

The molecular weights of P3HT with different molecular weights were determined by Gel Permeation Chromatography (GPC) (Agilent, PL 120 Plus, London, UK) with 0.1 mg/mL solution. The chemical structure of P3HT was recorded by Proton Nuclear Magnetic Resonance (^1^H NMR) (Bruker, 400 MHz, Zurich, Switzerland), with chloroform-d (CDCl_3_) as solvents. The Ultraviolet-visible (UV–Vis) and fluorescence were determined using the Lambda 750S (Perkin Elmer, Waltham, MA, USA) and Fluormax-4P (Horiba Jobin Yvon, Paris, France), respectively. The X-ray diffraction (XRD) measurement was determined by Smart Lab 9 KW (Rigaku, Tokyo, Japan). X-ray photoelectron spectrometry (XPS) was carried out by Escalab 250Xi (VG Scientific, Boston, MA, USA). Raman analysis and Fourier-transform infrared spectroscopy (FTIR) were conducted with Via-Reflex (Renishaw, London, UK) and TG209F3 (Bruker, Waltham, MA, USA), respectively. The microscopic morphology of the samples was examined by scanning electronic microscopy (SEM) (Hitachi, Regulus 8230, Tokyo, Japan) and electron microscopy (TEM) (Jeol, JEM-2100, Tokyo, Japan), respectively. The surface temperature was measured and the infrared thermal image of samples were taken with FLIR T1040 (FLIR, Boston, MA, USA). The thermal diffusivity (α) and the specific heat capacity (C_p_) were measured using LFA 467 (Netzsch, Munich, Germany) and TA Q2000 Instruments (TA, New Castle, DE, USA), respectively. The density (ρ) was achieved by the ratio of the mass and volume of the sample. The thermal conductivity was calculated according to the following formula:

(1) $$\kappa =\alpha \times C_{p}\times \rho$$

## 3. Results and Discussion

**Figure 1:**
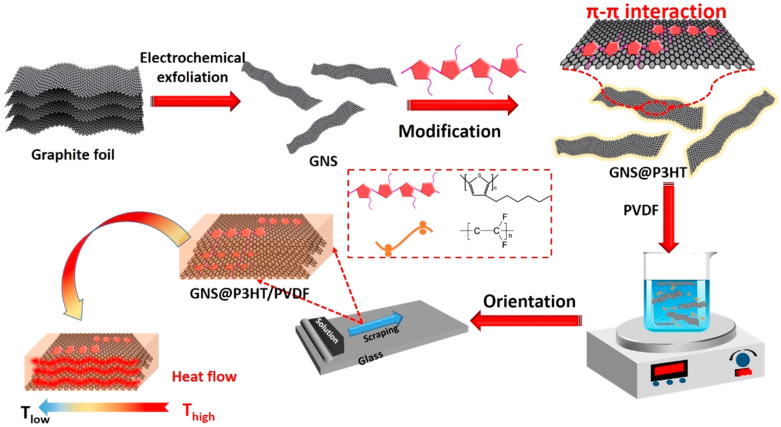
Schematic diagram of the preparation process of Graphene sheets @poly(3-hexylthiophene)/poly (vinylidene fluoride) (GNS@P3HT/PVDF).

In this paper, the effects of P3HT of four molecular weights on the thermal conductivity of the modified graphene composites was investigated. As shown in Figure 1, the preparation of GNS@P3HT/PVDF mainly includes three processes. Firstly, graphene was obtained by electrochemical exfoliation, which can conveniently obtain high- quality GNS [18]. The P3HT of four molecular weights was synthesized by the GRIM method, which could control effectively the molecular weight of P3HT. Secondly, the GNS@P3HT filler was obtained by π–π interaction between P3HT and GNS, which reduced the interfacial thermal resistance between GNS. Thirdly, due to the high aspect ratio of GNS, the GNS@P3HT/PVDF obtained has an ordered, stacked microstructure, achieved with the scraped film method. Long-range orderly heat transfer pathways were established by π–π interaction.

### 3.1. Structural Characterization of P3HT

**Figure 2:**
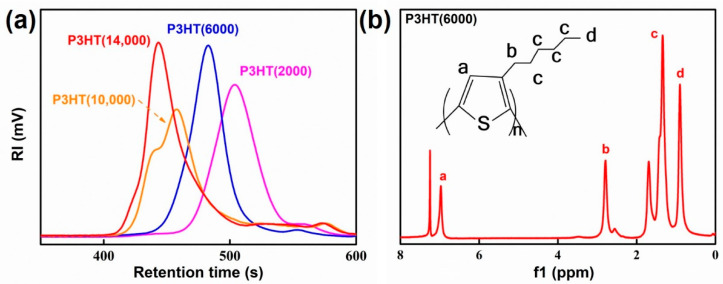
(**a**) Gel permeation chromatography (GPC) spectra of poly(3-hexylthiophene) (P3HT) with different molecular weights. (**b**) ^1^H nuclear magnetic resonance (NMR) spectra of P3HT (6000).

To explore the effects of different molecular weights of P3HT of the modified graphene composite on thermal conductivity, P3HT with four molecular weights was synthesized by changing the reaction temperature and the amount of Ni(dppp)Cl_2_ catalyst in the GRIM method [16]. Figure 2a and Table 1 show the GPC spectra at different molecular weights of P3HT and the synthesis conditions of corresponding molecular weights, respectively. The peaks of four molecular weights of P3HT were all single peaks, which indicated that the obtained P3HT polymers were homopolymers. In addition, the synthesis conditions of parallel experiments with three molecular weights of P3HT were obtained by measuring the molecular weight of P3HT in Table 1. The P3HT with three molecular weights were prepared by increasing the reaction temperature of the system under the unchanged condition of the molar ratio of 2,5-dibromo-3-hexylthiophene monomer of format reagent and catalyst. The P3HT with a molecular weight of 14000 g/mol was synthesized by reducing the amount of catalyst. When the amount of catalyst was too large, there were more active sites, which led to a lower molecular weight [19,20].

The ^1^H NMR spectra in Figure 2b and Figure S2 show P3HT with four molecular weights, respectively. The peak at δ = 6.97 ppm was attributed to the hydrogen proton of the thiophene ring (Ar–H, a); the peak at δ = 2.79 ppm corresponded to the hydrogen proton (Ar–CH_2_–, b) on the methylene group attached to the thiophene ring; the peaks at δ = 0.8–1.0 ppm and 1.34–1.69 ppm can be attributed to the hydrogen proton (–CH_3_, d) on the terminal methyl group of the substituent and other methylenes on the side chain of the thiophene ring (–(CH_2_)_4_–, c), respectively [21,22,23,24,25]. Although the positions of the individual peaks of four molecular weights of P3HT are divergent, they all basically shifted near their respective peaks. Hence, four molecular weights of P3HT were prepared successfully.

### 3.2. Fabrication of GNS@P3HT(X) and GNS@P3HT(X)/PVDF Membranes

**Figure 3:**
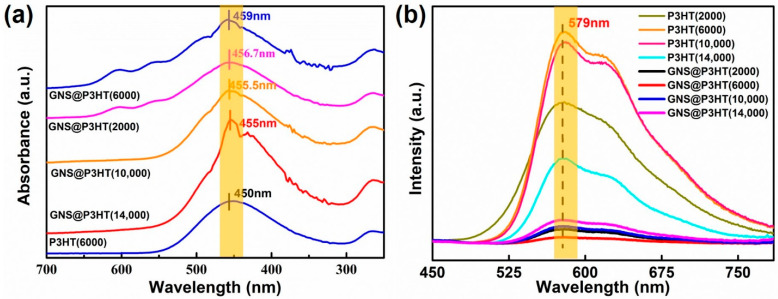
(**a**) Ultraviolet-visible (UV–Vis) absorption spectra of Graphene sheets @poly(3-hexylthiophene) (GNS@P3HT) with different molecular weights (0.001 g/mL). (**b**) Fluorescence spectra (λ = 400 nm) of GNS@P3HT with different molecular weights (0.001 g/mL).

In order to verify that GNS modified by P3HT with different molecular weights were realized by π–π interaction, the interaction between P3HT and GNS was characterized by UV–Visible and fluorescence spectroscopy. The UV–Vis spectra of P3HT and GNS@P3HT with different molecular weights at a concentration of 0.001 g/mL is shown in Figure S4 and Figure 3a. The UV–Vis absorption peaks of P3HT with different molecular weights were all around 450 nm in Figure S4. Since P3HT (6000) has the highest absorption peak intensity at 450 nm, it was chosen as the comparison group with GNS@P3HT. In Figure 3a, GNS@P3HT with different molecular weights have only one absorption peak in chloroform, which indicated that GNS@P3HT was not simply mixed but forms a single unit in solution by π–π interaction. In addition, compared with the spectrum of P3HT (6000), the positions of the absorption peaks of GNS@P3HT were redshifted by at least 5 nm. The occurrence of redshift was mainly due to the π–π interaction between P3HT and GNS, which caused a change of the surface charge of P3HT [26,27,28]. Moreover, compared with the other three, GNS@P3HT (6000) had the largest redshift distance, which was probably due to the strongest adhesion to the GNS surface with P3HT(6000) in this state, which produced a stronger degree of conjugation with GNS [29,30].

Under the conditions of sample concentration of 0.001 g/mL and excitation wavelength of 400 nm, the fluorescence spectra of P3HT and GNS@P3HT with different molecular weights were obtained, as shown in Figure 3b. The wide diffraction peaks of P3HT with different molecular weights appear around 579 nm, which indicates that the electrons in the outer layers of P3HT are induced to transition from the ground state to a higher energy level, and then back to a lower energy level, thus emitting visible fluorescence [31]. With the increase of molecular weight of P3HT, the corresponding fluorescence intensity first increases and then decreases. This was because P3HT with a low molecular weight is easily dispersed in solution and forms stronger intra-chain or inter-chain interaction aggregates, which shows more color-emitting groups. However, the long chains did not stretch easily in the solvent for the high molecular weight of P3HT and this led to entanglement and aggregation [29,30,32,33,34,35]. Compared with the fluorescence of P3HT, the fluorescence of GNS@P3HT was significantly quenched. Considering that there was no serious aggregation of P3HT, it indicated that an electron transfer complex was formed between P3HT and GNS by π–π interaction. The electrons on P3HT were greatly restricted by the motion and cannot transition between energy levels, which leads to the quenching of the fluorescence of P3HT. [31,35,36,37,38,39]. Therefore, the movement of electrons on the surface of P3HT was limited by the π–π interaction of GNS, which leads to the fluorescence quenching of GNS@P3HT. In addition, the fluorescence intensity quenching of P3HT (6000) was the strongest, which indicates that P3HT (6000) and GNS had the strongest π–π interaction, which made the electron transfer on P3HT (6000) the most difficult. The π–π interaction between P3HT and GNS in GNS@P3HT was confirmed by the UV–Vis spectrum and fluorescence analysis.

**Figure 4:**
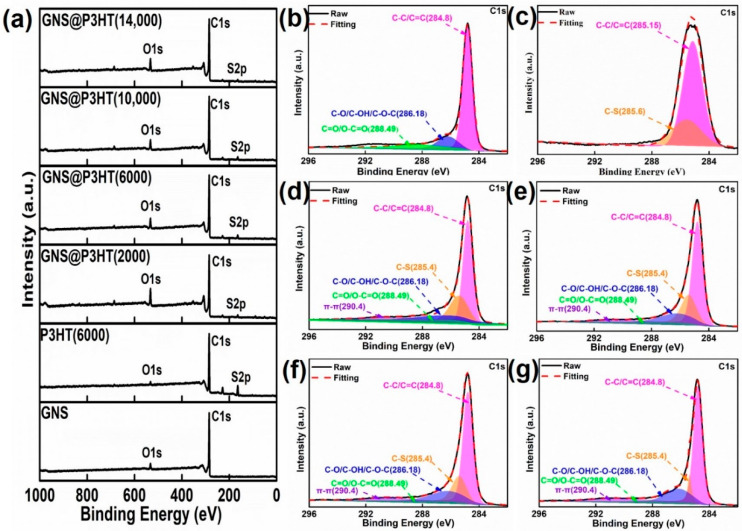
(**a**) X-ray photoelectron spectrometry (XPS) spectra of Graphene sheets (GNS), P3HT (6000) and GNS@P3HT with different molecular weights. (**b**) C1s spectrum of XPS of GNS. (**c**) C1s spectrum of XPS of P3HT (6000). (**d**) C1s spectrum of XPS of GNS@P3HT (2000). (**e**) C1s spectrum of XPS of GNS@P3HT (6000). (**f**) C1s spectrum of XPS of GNS@P3HT (10,000). (**g**) C1s spectrum of XPS of GNS@P3HT (14,000).

XPS spectroscopy can show the changes of surface chemical states of GNS modified by P3HT with different molecular weights (see Figure 4). Compared with GNS and P3HT (6000), the spectra of GNS@P3HT with different molecular weights showed S_2p_ peaks, which indicated the presence of a sulfur element. The three deconvoluted peaks of GNS correspond to C–C/C=C (284.80 eV), C–O/C–OH/C–O–C (286.18 eV), and C=O/O–C=O (288.49 eV), respectively, in Figure 4b [40,41]. In Figure 4c, the deconvoluted peaks of P3HT (6000) are attributed to C–C/C=C (285.15 eV) and C–S (285.60 eV), respectively [42,43]. According to Figure 4d–g, the deconvoluted peaks of 284.80 eV, 285.40 eV, 286.18 eV, 288.49 eV, and 290.4 eV in the C1s spectra of GNS@P3HT with different molecular weights are ascribed to C–C/C=C, C–S, C–O/C–OH/C–O–C, C=O/O–C=O and π–π, respectively [40,42,43]. The GNS@P3HT has characteristic peaks of GNS and P3HT, which indicates that P3HT was loaded on the GNS surface. The relative atomic percentage of the samples were analyzed by XPS in Table 2. The relative atomic ratios of S/C of GNS and P3HT (6000) were 0% and 9.52%, respectively. The relative atomic ratios of S/C in GNS@P3HT with different molecular weights were 3.29%, 3.47%, 2.52%, and 2.01%, respectively. In addition, according to the TG diagram in Figure S5, the loadings of P3HT of GNS@P3HT (2000), GNS@P3HT (6000), GNS@P3HT (10,000), and GNS@P3HT (14,000) were 14.97 wt%, 16.89 wt%, 12.34 wt%, and 9.14 wt%, respectively.

**Figure 5:**
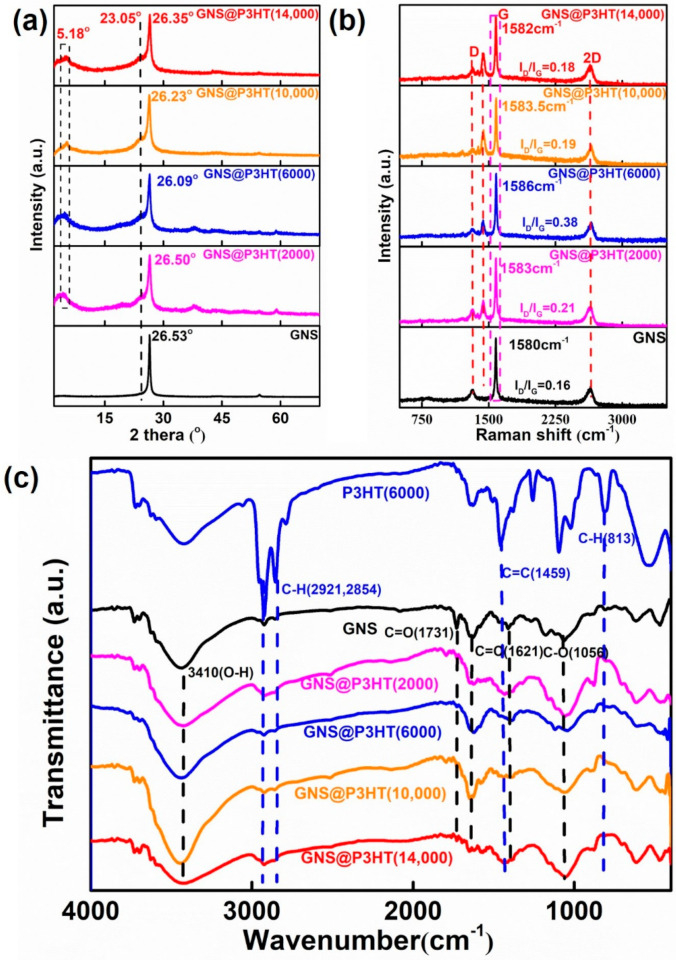
(**a**) X-ray diffraction (XRD) spectra of GNS and GNS@P3HT with different molecular weights. (**b**) Raman spectra of GNS and GNS@P3HT with different molecular weights. (**c**) Fourier-transform infrared (FTIR) spectra of GNS@P3HT with different molecular weights.

Figure 5a showed the XRD spectra of GNS and P3HT@GNS with different molecular weights. The diffraction peak of 26.53° corresponded to the (002) crystal plane in the GNS spectrum; the diffraction peaks at 5.18° and 23.05° were assigned to the (100) and (300) crystal planes of P3HT, respectively, while the GNS@P3HT spectrum has characteristic diffraction peaks of both P3HT and GNS [44]. Compared with the (002) crystal plane of GNS, the intensity of the (002) crystal plane of GNS@P3HT with different molecular weights was reduced and broadened. In addition, the leftward shift of the (002) crystal plane of P3HT@GNS indicated that the layer spacing of GNS increased, which also indicated that P3HT had been loaded on the surface of GNS.

From the Raman spectrum of GNS@P3HT in Figure 5b, the peak at 1425 cm^−1^ belonged to the C=C symmetric stretching peak on the thiophene ring in P3HT [45,46,47], and the peaks at 1309 cm^−1^ and 1582 cm^−1^ were attributed to the D band and G band of GNS, respectively. The I_D_/I_G_ (intensity ratio of the D band and G band) of GNS/P3HT (2000), GNS/P3HT (6000), GNS/P3HT (10,000), and GNS/P3HT (14,000) increased to 0.21, 0.38, 0.19, and 0.18, respectively, when GNS has an I_D_/I_G_ of 0.16. By comparing the I_D_/I_G_, the results indicated that the defect was introduced on the GNS by surface modification of P3HT; however, the non-covalent modification of P3HT had slightly damaged the GNS of GNS@P3HT. Compared with the GNS, the positions of the G band of GNS@P3HT were blueshifted. P3HT was attached to the GNS surface through π–π interactions, which changed the charge distribution on the surface of GNS. Thus, the vibration of GNS@P3HT required higher energy and shifted the G band to a higher frequency [48,49].

The characteristics group of GNS@P3HT with different molecular weights was determined by FTIR spectra, as shown in Figure 5c. The characteristic peak at 3410 cm^−1^ was due to O–H bending vibration, and the characteristic peaks at 1731 cm^−1^, 1621 cm^−1^, and 1056 cm^−1^ were attributed to the C=C stretching vibration peak and C–O stretching vibration peak, respectively, in the GNS spectrum of Figure 5c [50]. In the spectrum of P3HT (6000), the characteristic peaks at 2921 cm^−1^ and 2854 cm^−1^ contributed to the stretching vibration peak of C–H on the thiophene ring; the peaks near 1459 cm^−1^ and 813 cm^−1^ corresponded to the stretching vibration peak of C=C and the out-of-plane bending vibration peak of C–H on the thiophene ring, respectively. Compared with P3HT (6000) and GNS spectra, the GNS@P3HT with different molecular weights has the characteristics group of P3HT and GNS, which indicated that P3HT was loaded on the surface of GNS.

**Figure 6:**
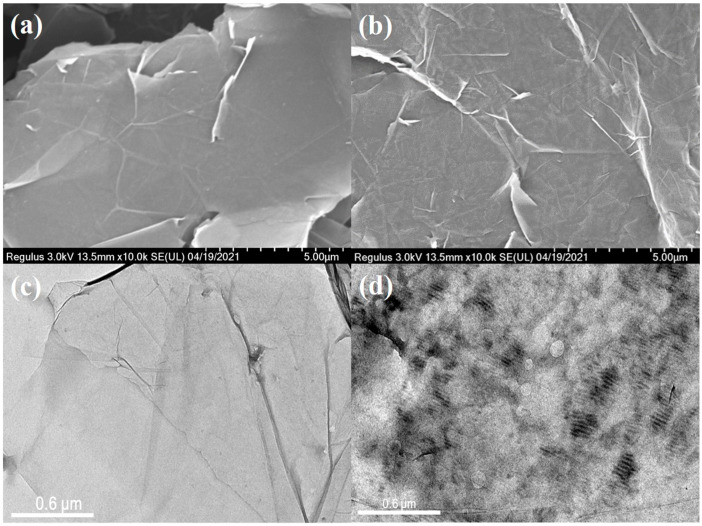
(**a**) Scanning electron microscopy (SEM) images of GNS. (**b**) SEM images of GNS@P3HT (6000). (**c**) Transmission electron microscopy (TEM) images of GNS. (**d**) TEM images of GNS@P3HT (6000).

The SEM and TEM have been utilized to further investigate the morphology of GNS and GNS@P3HT. Figure 6a,b shows the SEM images of GNS and GNS@P3HT (6000), respectively. The GNS has a smooth surface without damage and a large size, while the GNS@P3HT (6000) also has a large size structure but its surface is rough with small protruding particles. Therefore, P3HT (6000) was loaded on the surface of the GNS. In Figure 6c, the GNS, due to electrochemical exfoliation, has a typical folded structure with fewer layers, a transparent, flat surface, and a large spreading chord ratio. Compared with Figure S6, the entire surface of GNS modified by P3HT (6000) was uniformly covered with a gray organic material texture and has a distinctly deep strip texture in some areas in Figure 6d; therefore, P3HT was not formed during deposition process, but formed in solution [41,45].

**Figure 7:**
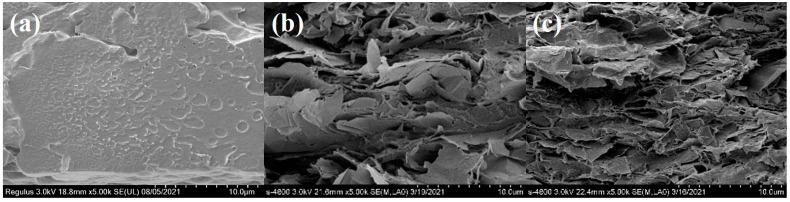
SEM images of GNS/PVDF and GNS@P3HT/PVDF membranes: (**a**) PVDF; (**b**) 20 wt% GNS/PVDF; (**c**) 20 wt% GNS@P3HT (6000)/PVDF.

SEM could be used to characterize the internal microstructure of the membrane, as shown in Figure 7. The cross-section of pure PVDF was relatively flat and there were a few micro pits left by the process of heating and removing the solvent during the preparation of membranes in Figure 7a. In Figure 7b, GNS was oriented along the horizontal direction in membranes, but its distribution was uneven with a large agglomeration phenomenon. The interface between GNS and PVDF was clearly distinguished, which made the interface less compatible. Compared with Figure S7, GNS@P3HT (6000) has a good dispersion in PVDF without obvious agglomeration. GNS@P3HT (6000)/PVDF had a denser stacking without an obvious cavity and apparent interface separation in the cross section. The P3HT (6000) loading on the surface of GNS could reduce the interface thermal resistance between GNS, thereby reducing the scattering of phonon transfer between GNS, which facilitated the formation of the heat conduction pathway. Therefore, the thermal conductivity of 20 wt% GNS@P3HT (6000)/PVDF will be greatly improved, which can be verified by the thermal conductivity test.

### 3.3. Thermal Properties of GNS@P3HT/PVDF Membranes

**Figure 8:**
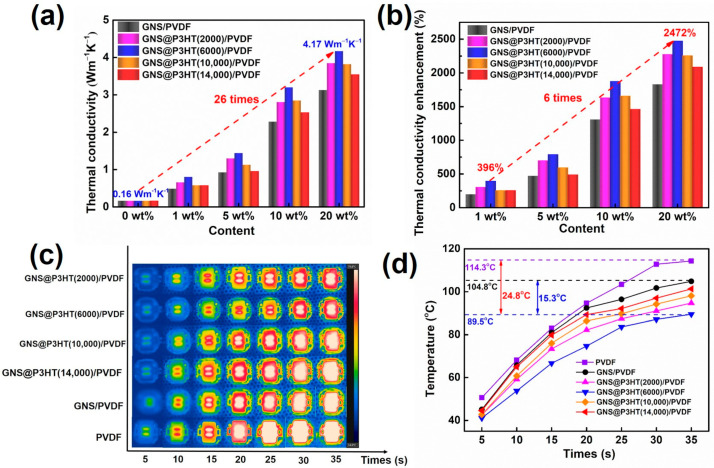
(**a**) The thermal conductivity of GNS@P3HT/PVDF membranes with different filler loadings. (**b**) Thermal conductivity enhancement of GNS@P3HT/PVDF with different filler loadings. (**c**) The infrared thermal images of the light-emitting diode (LED) chips integrated with PVDF, GNS/PVDF, and GNS@P3HT/PVDF. (**d**) The surface temperature curves of LED chips integrated with P3HT@GNS/PVDF in 35 s.

To verify the effect of P3HT with different molecular weights on the modified GNS, the effect was verified by the in-plane thermal conductivity of the GNS@P3HT/PVDF membrane. The thermal conductivity of PVDF membranes improved with the increase of filler content, as shown in Figure 8a, Tables S1 and S2. The sequence of the influence of P3HT with different molecular weights on the thermal conductivity of GNS@P3HT membranes was as follows: GNS@P3HT (6000)/PVDF, GNS@P3HT (2000)/PVDF, GNS@P3HT (10,000)/PVDF, GNS@P3HT (14,000) /PVDF, and GNS/PVDF. The in-plane thermal conductivity of the GNS@P3HT (6000)/PVDF membrane was 4.17 W m^−1^ K^−1^ at a filler loading of 20 wt%, which was 26 times higher than that of pure PVDF. The thermal conductivity of the GNS@P3HT membrane was significantly greater than that of the GNS membrane. The GNS modified by P3HT with different molecular weights could have been uniformly dispersed and oriented in PVDF, which formed a dense thermal conductivity pathway of PVDF membranes [37]. Simultaneously, the interface thermal resistance between GNS was reduced, which, in turn, improved the thermal conductivity of the membrane. In addition, the thermal conductivity of GNS@P3HT (6000)/PVDF was the highest relative to the modified graphene membranes of other P3HT molecular weights. Compared with the other three modified molecular weights, the P3HT chain with a molecular weight of 6000 g/mol formed a highly matched structure on the surface of GNS when the chain interacts with GNS through π–π interaction, which was beneficial to the transfer of phonons between GNS sheets and reduced the scattering during phonon transfer. However, the excessive molecular chains might be stacked in a somewhat disorderly manner on the surface of GNS, resulting in a weak interaction between P3HT and GNS in this region, which will greatly hinder phonon transmission through P3HT to nearby GNS. Therefore, the thermal conductivities of composites of P3HT modified graphene with different molecular weights were also different. In addition, the thermal percolation threshold of PVDF composites with graphene and GNS@P3HT fillers was around 5 wt% (see Figure S8). Below the percolation threshold, the thermal conductivity of PVDF membranes increased slowly. The GNS or GNS@P3HT was dispersed in PVDF without constructing a heat transfer pathway, which produce severe phonon scattering and high interfacial thermal resistance [51,52]. Above the thermal percolation threshold, the thermal conductivity of PVDF membranes increased rapidly. The filler formed an in-plane heat transfer pathway in the PVDF; at this time, the thermal conductivity of the filler governed the thermal conductivity of PVDF membranes [52,53]. Simultaneously, in order to compare the efficiency of different modified fillers on the thermal conductivity of the substrate, thermal conductivity enhancement efficiency (TCE) can be compared:

(2) $$\mathrm{TCE}=\frac{\kappa_{c}-\kappa_{p}}{\kappa_{p}}\;\times 100\%$$

where κ_c_ and κ_p_ represent the thermal conductivity of membranes and pure PVDF, respectively.

As shown in Figure 8b, the TCE of GNS@P3HT (6000)/PVDF (20 wt%) composite was 2472%, which was significantly higher than that of the other fillers and six times that of GNS@P3HT (6000)/PVDF (1 wt%). These results indicated that the stronger π–π interactions between P3HT (6000 g/mol) and GNS served to improve the dispersibility of modified GNS, which prepared the stable organic reagent dispersions of GNS with a stabilizer of P3HT and reduced the interface thermal resistance between fillers [37,54]. The composites of GNS modified with P3HT of a molecular weight of 6000 g/mol has the highest thermal conductivity.

In order to visually evaluate the thermal conductivity of the composite of P3HT modified GNS with different molecular weights, the surface temperature of the LED lamp, with the membrane glued to the base of the commercialized LED lamp using thermally conductive silver glue, was monitored by infrared thermography within a 35 s period, and the results are shown in Figure 8c,d. The temperature of the LED lamp on the GNS@ P3HT (6000)/PVDF was 89.5 °C at 35 s, which was 24.8 °C and 15.3 °C lower than that of the pure PVDF and GNS/PVDF, respectively. Due to the good interaction between P3HT (6000) and GNS, which significantly reduced the interfacial thermal resistance of the composite, the membrane of P3HT (6000) modified GNS has the highest thermal conductivity out of GNS@ P3HT/PVDF.

## 4. Conclusions

In this paper, modified GNS was prepared by π–π interaction of P3HT with different molecular weights, and the orientation of the modified GNS within the PVDF membrane was realized using a scraped membrane method. GNS@P3HT reduced the interfacial thermal resistance between GNS, which facilitated the formation of a heat conduction pathway in GNS@P3HT/PVDF. When GNS was modified by P3HT with different molecular weights, the membrane of modified GNS by P3HT with a molecular weight of 6000 was found to have the highest thermal conductivity. The thermal conductivity of the GNS@P3HT/PVDF membrane was 4.17 W m^−1^ K^−1^ with a 20 wt% addition of GNS@P3HT (6000), which was 26 times that of pure PVDF. This conclusion has not only enriched understanding of methods for non-covalent modification of thermal conductivity fillers but also extended the potential for surface modifications to other substances.

## Figures and Tables

**Table 1:** Synthesis conditions of P3HT with different molecular weights.

M_n_ (g/mol)	Molar Ratio of CH_3_BrMg/C_10_H_14_Br_2_S	Molar Ratio of C_10_H_14_Br_2_S/Ni(dppp)Cl_2_	T/°C	t/h
2000	1.1:1	100:1	25	2
6000	1.1:1	100:1	30	2
10,000	1.1:1	100:1	40	2
14,000	1.1:1	125:1	40	2

**Table 2:** Relative atomic percentage of GNS, P3HT(6000), and GNS@P3HT samples.

Sample	C (%)	S (%)	S/C (%)
GNS	94.66	-	-
P3HT (6000)	89.1	8.48	9.52
GNS@P3HT (2000)	79.64	2.62	3.29
GNS@P3HT (6000)	89.44	3.1	3.47
GNS@P3HT (10,000)	84.49	2.13	2.52
GNS@P3HT (14,000)	88.73	1.78	2.01

## Data Availability

Data are available on request from corresponding authors.

## Supplementary Materials

The following are available online at https://www.mdpi.com/article/10.3390/membranes11110895/s1, Figure S1: Synthetic routes of P3HT by the GRIM method; Figure S2: Optical images of 25 wt% GNS@P3HT/PVDF in (a,b); Figure S3: 1H NMR spectra of (a) P3HT (2000), (b) P3HT (10,000), and (c) P3HT (14,000); Figure S4: UV–Vis spectra of P3HT at different molecular weights; Figure S5: TGA curves of GNS@P3HT with different molecular weights; Figure S6: TEM images of (a) GNS@P3HT (2000), (b) GNS@P3HT (10,000), and (c) GNS@P3HT (14,000); Figure S7: SEM images of (a) 20 wt% GNS@P3HT (2000)/PVDF, (b) 20 wt% GNS@P3HT (10,000)/PVDF, and (c) 20 wt% GNS@P3HT (14,000)/PVDF; Table S1: Density of the GNS/PVDF and GNS@P3HT/PVDF membrane with different filler mass fractions; Table S2: Physical properties of the GNS/PVDF and GNS@P3HT/PVDF membrane with different filler mass fractions; Figure S8: Thermal conductivity of the PVDF membranes with GNS and GNS@P3HT fillers with different filler loading.
