# Supplementary material for: Interfacial Modulation of Graphene by Polythiophene with Controlled Molecular Weight to Enhance Thermal Conductivity
Source: Membranes (Basel). 2021 Nov 19;11(11):895. doi: 10.3390/membranes11110895 (PMC8625024; doi:10.3390/membranes11110895)
Supplement: Supplementary file 1 [file membranes-11-00895-s001.zip › membranes-1431660-supplementary.pdf]

membranes

Article

Interfacial Modulation of Graphene by Polythiophene with Controlled Molecular Weight to Enhance Thermal Conductivity

Ya Li, Yu Wang, Peng Chen, Ru Xia, Bin Wu * and Jiasheng Qian *

Key Laboratory of Environment-Friendly Polymeric Materials of Anhui Province, School of Chemistry & Chemical Engineering, Anhui University, Hefei 230601, China; c18101004@stu.ahu.edu.cn (Y.L.); c19301127@stu.ahu.edu.cn (Y.W.); chenpeng@ahu.edu.cn (P.C.); 96047@ahu.edu.cn (R.X.)

* Correspondence: lwbin@ahu.edu.cn (B.W.); qianjsh@ahu.edu.cn (J.Q.)

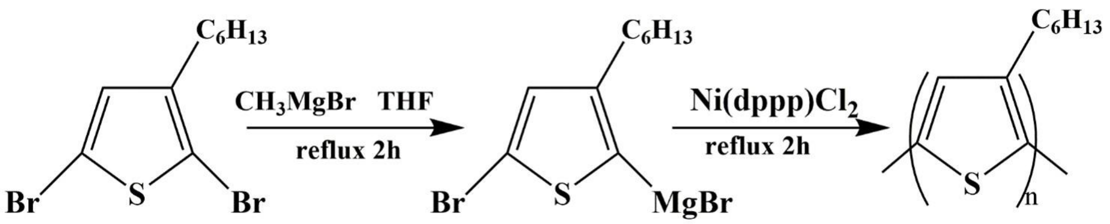

Figure S1. Synthetic routes of P3HT by GRIM method.

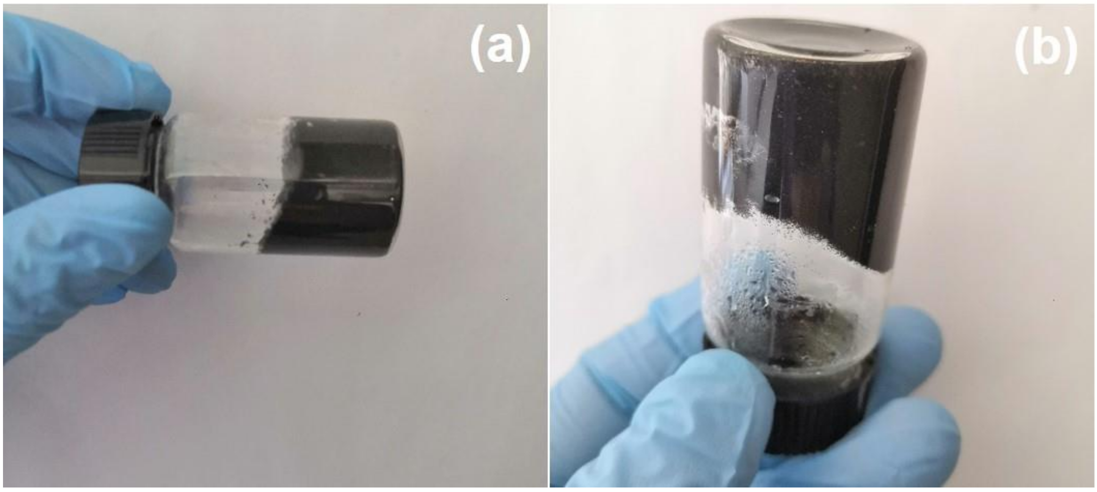

Figure S2. Optical images of 25 wt% GNS@P3HT/PVDF in (**a**) and (**b**).

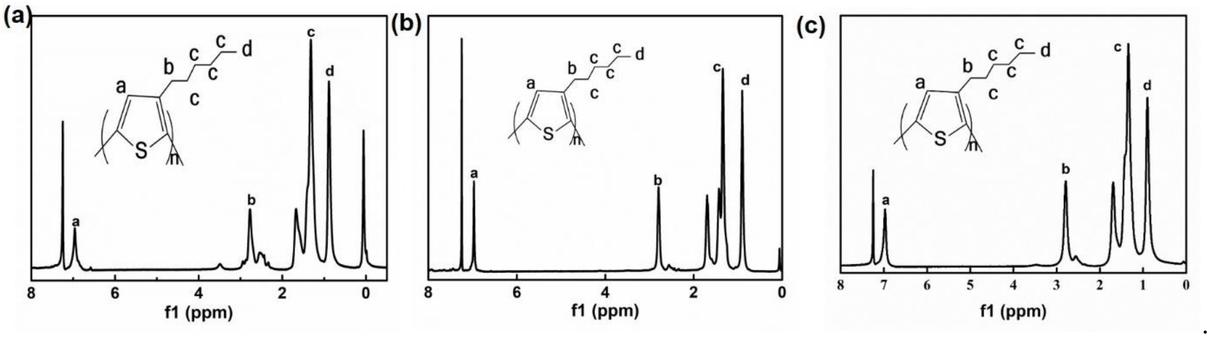

Figure S3. ^{1}H NMR spectra of (**a**) P3HT(2000), (**b**) P3HT(10,000) and (**c**) P3HT(14,000).

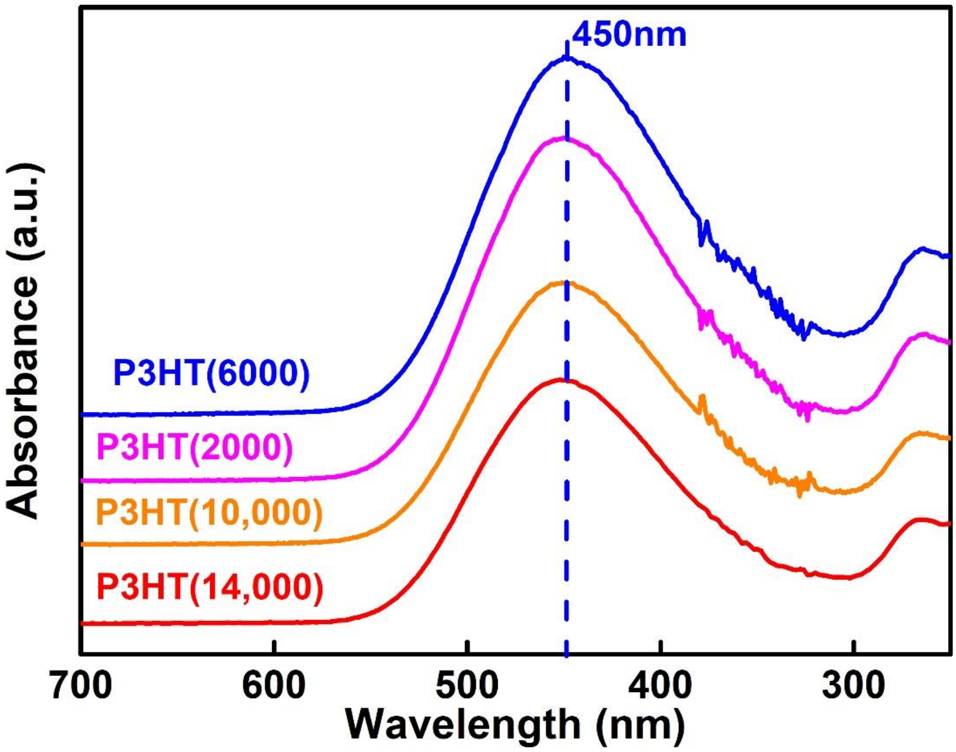

Figure S4. UV-vis spectra of P3HT at different molecular weights.

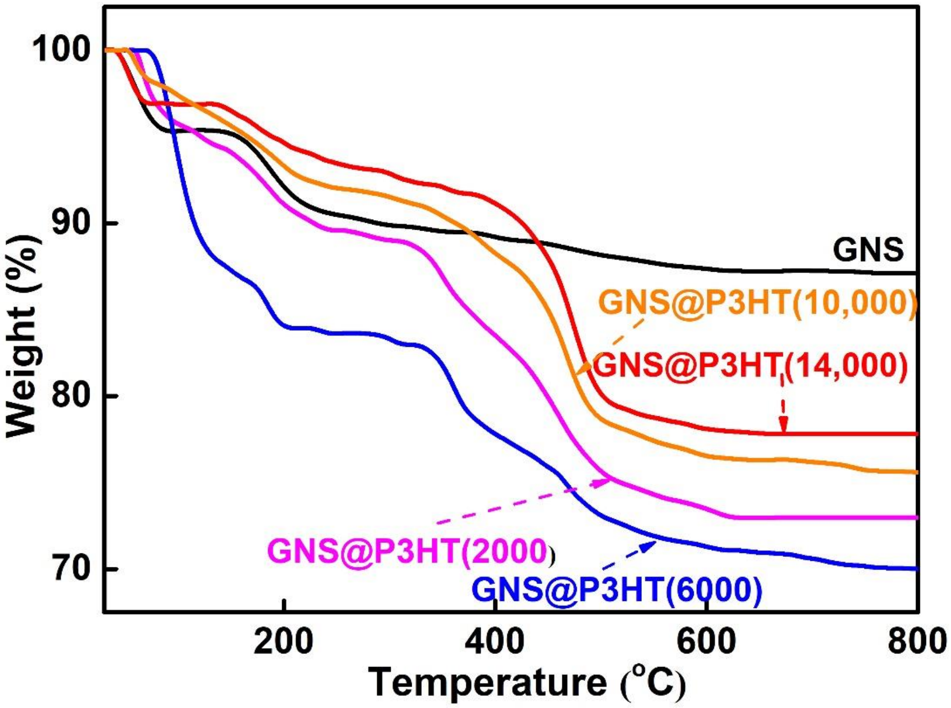

Figure S5. TGA curves of GNS@P3HT with different molecular weights.

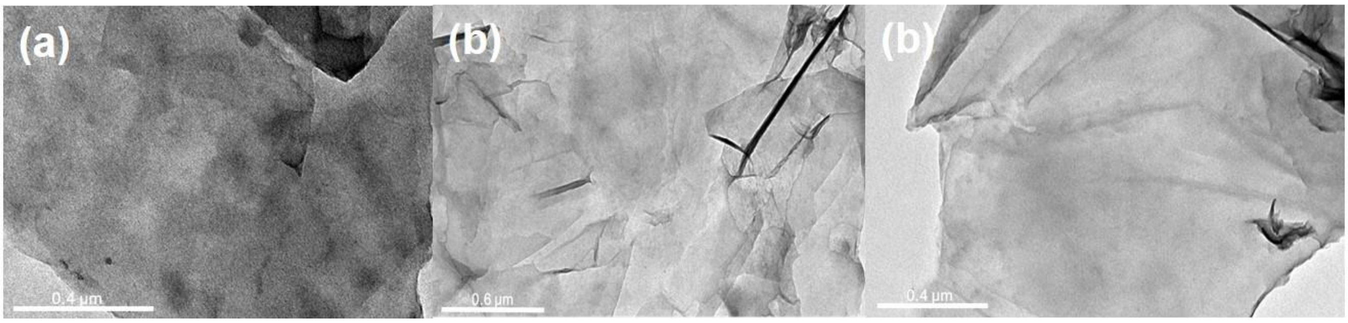

Figure S6. TEM images of (**a**) GNS@P3HT(2000), (**b**) GNS@P3HT(10,000) and (**c**) GNS@P3HT(14,000).

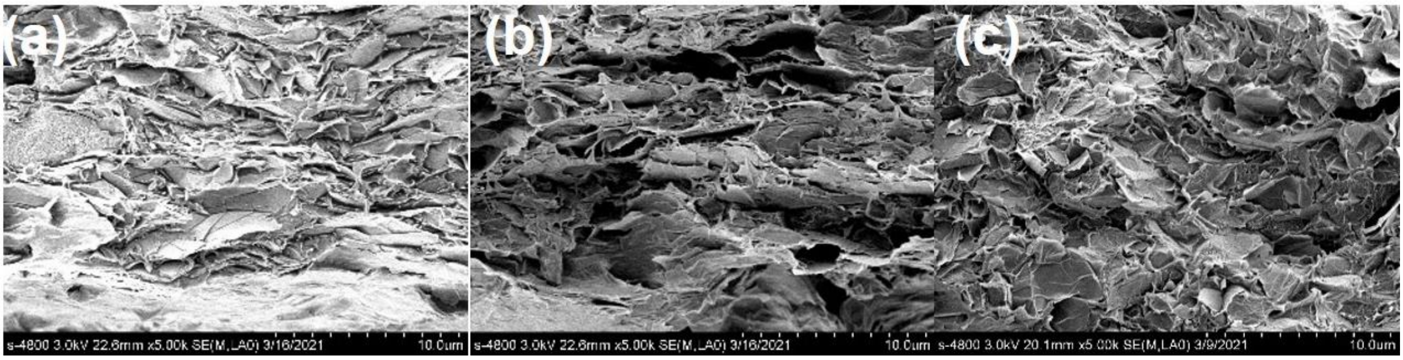

Figure S7. SEM images of (**a**) 20 wt% GNS@P3HT(2000)/PVDF, (**b**) 20 wt% GNS@P3HT(10,000)/PVDF and (**c**) 20 wt% GNS@P3HT(14,000)/PVDF.

Table S1. Density of the GNS/PVDF and GNS@P3HT/PVDF membrane with different filler mass fractions**.**

Mass fraction	Mass (g)	Height (mm)	Density (g cm^{-3})
	0.022	0.083	1.64
PVDF	0.024	0.086	1.75
	0.021	0.085	1.52
	0.020	0.078	1.61
1wt%GNS/PVDF	0.021	0.071	1.84
	0.023	0.079	1.77
	0.027	0.094	1.78
1wt%GNS@P3HT(2000)/PVDF	0.026	0.091	1.77
	0.028	0.098	1.76
	0.023	0.084	1.72
1wt%GNS@P3HT(6000)/PVDF	0.025	0.089	1.74
	0.024	0.086	1.75
	0.024	0.085	1.73
1wt%GNS@P3HT(10,000)/PVDF	0.023	0.083	1.76
	0.025	0.089	1.73
	0.023	0.081	1.74
1wt%GNS@P3HT(14,000)/PVDF	0.024	0.084	1.75
	0.025	0.087	1.75
	0.026	0.089	1.78
5wt%GNS/PVDF	0.027	0.095	1.77
	0.026	0.092	1.74
5wt%GNS@P3HT(2000)/PVDF	0.021	0.071	1.80
	0.023	0.079	1.77

	0.022	0.076	1.79
	0.021	0.073	1.75
5wt%GNS@P3HT(6000)/PVDF	0.021	0.075	1.73
	0.020	0.071	1.75
	0.024	0.085	1.75
5wt%GNS@P3HT(10,000)/PVDF	0.024	0.087	1.73
	0.023	0.082	1.77
	0.025	0.087	1.75
5wt%GNS@P3HT(14,000)/PVDF	0.024	0.085	1.75
	0.023	0.082	1.77
	0.018	0.063	1.81
10wt%GNS/PVDF	0.019	0.067	1.80
	0.020	0.068	1.81
	0.024	0.081	1.79
10wt%GNS@P3HT(2000)/PVDF	0.025	0.087	1.77
	0.024	0.085	1.76
	0.022	0.077	1.76
10wt%GNS@P3HT(6000)/PVDF	0.022	0.079	1.73
	0.023	0.081	1.76
	0.023	0.081	1.79
10wt%GNS@P3HT(10,000)/PVDF	0.024	0.085	1.78
	0.025	0.089	1.77
	0.025	0.089	1.75
10wt%GNS@P3HT(14,000)/PVDF	0.027	0.098	1.74
	0.026	0.092	1.73
	0.025	0.086	1.81
20wt%GNS/PVDF	0.022	0.078	1.73
	0.024	0.081	1.83
	0.022	0.073	1.85
20wt%GNS@P3HT(2000)/PVDF	0.022	0.075	1.83
	0.021	0.071	1.84
	0.025	0.085	1.80
20wt%GNS@P3HT(6000)/PVDF	0.024	0.081	1.81
	0.026	0.088	1.82
	0.021	0.071	1.85
20wt%GNS@P3HT(10,000)/PVDF	0.023	0.079	1.83
	0.021	0.073	1.81
	0.023	0.081	1.79
20wt%GNS@P3HT(14,000)/PVDF	0.026	0.089	1.77
	0.025	0.085	1.80

Table S2. Physical properties of the GNS/PVDF and GNS@P3HT/PVDF membrane with different filler mass fractions.

Mass fraction	Density (g cm^{-3})	Specific heat capacity (J $g^{-1}K^{-1}$)	Thermal diffusivity (mm^2 s^{-1})	Thermal conductivity (W m^{-1} K^{-1})
	1.645	1.153	0.087	0.165
PVDF	1.753	1.144	0.081	0.162
	1.514	1.137	0.093	0.160
1wt%GNS/PVDF	1.609	1.100	0.261	0.462

	1.844	1.062	0.256	0.501
	1.766	1.122	0.245	0.485
1wt%GNS@P3HT(2000)/PVDF	1.779	1.094	0.344	0.670
	1.766	1.125	0.330	0.656
	1.760	1.073	0.342	0.646
1wt%GNS@P3HT(6000)/PVDF	1.723	1.103	0.418	0.794
	1.737	1.108	0.428	0.824
	1.752	1.107	0.414	0.803
1wt%GNS@P3HT(10,000)/PVDF	1.733	1.044	0.328	0.593
	1.755	1.059	0.311	0.578
	1.726	1.034	0.319	0.569
1wt%GNS@P3HT(14,000)/PVDF	1.739	1.087	0.310	0.586
	1.753	1.111	0.291	0.567
	1.746	1.097	0.301	0.576
5wt%GNS/PVDF	1.779	1.113	0.467	0.925
	1.766	0.950	0.528	0.886
	1.739	0.940	0.590	0.964
5wt%GNS@P3HT(2000)/PVDF	1.803	1.148	0.639	1.323
	1.770	1.111	0.6591	1.296
	1.787	1.096	0.6552	1.283
5wt%GNS@P3HT(6000)/PVDF	1.753	1.106	0.743	1.441
	1.727	1.094	0.751	1.419
	1.745	1.091	0.773	1.471
5wt%GNS@P3HT(10,000)/PVDF	1.754	1.022	0.628	1.126
	1.731	1.043	0.650	1.174
	1.769	0.919	0.644	1.047
5wt%GNS@P3HT(14,000)/PVDF	1.754	1.054	0.517	0.955
	1.747	1.030	0.535	0.962
	1.769	1.022	0.521	0.942
10wt%GNS/PVDF	1.814	1.181	1.078	2.310
	1.796	1.126	1.127	2.279
	1.805	1.141	1.081	2.227
10wt%GNS@P3HT(2000)/PVDF	1.794	1.077	1.451	2.804
	1.772	1.026	1.431	2.603
	1.758	1.055	1.512	2.805
10wt%GNS@P3HT(6000)/PVDF	1.756	1.148	1.586	3.198
	1.732	1.139	1.643	3.241
	1.764	1.158	1.527	3.119
10wt%GNS@P3HT(10,000)/PVDF	1.794	1.041	1.536	2.869
	1.780	1.048	1.526	2.847
	1.765	1.043	1.532	2.821
10wt%GNS@P3HT(14,000)/PVDF	1.752	0.981	1.471	2.528
	1.739	0.968	1.487	2.504
	1.725	0.993	1.493	2.558
20wt%GNS/PVDF	1.810	1.119	1.544	3.125
	1.733	1.116	1.633	3.158
	1.832	1.127	1.503	3.104
20wt%GNS@P3HT(2000)/PVDF	1.847	1.170	1.796	3.881
	1.829	1.157	1.823	3.857
	1.838	1.148	1.804	3.805
20wt%GNS@P3HT(6000)/PVDF	1.804	1.057	2.186	4.169

	1.811	1.051	2.118	4.030
	1.825	1.073	2.13	4.171
20wt%GNS@P3HT(10,000)/PVDF	1.851	1.057	2.019	3.952
	1.826	1.042	2.011	3.826
	1.809	1.023	1.983	3.670
20wt%GNS@P3HT(14,000)/PVDF	1.794	0.992	2.053	3.655
	1.772	1.004	1.956	3.482
	1.802	1.017	1.965	3.599

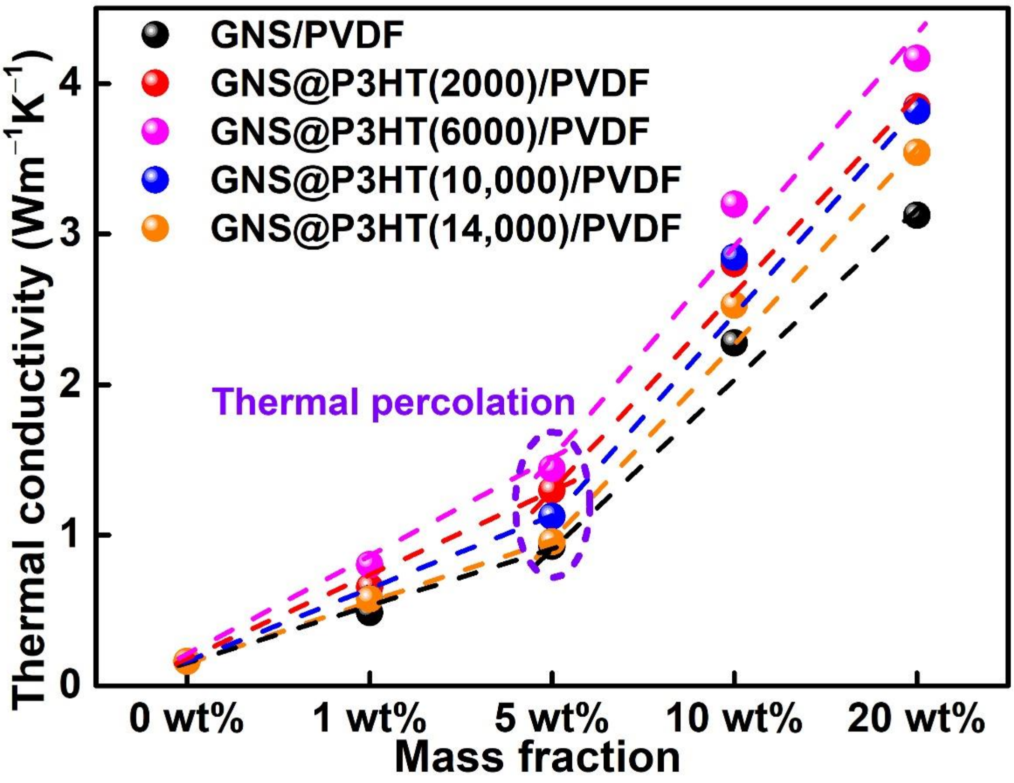

Figure S8. Thermal conductivity of the PVDF membranes with GNS and GNS@P3HT fillers with different filler loading.
